# Modulation of Thyroid Hormone-Dependent Gene Expression in *Xenopus laevis* by INhibitor of Growth (ING) Proteins

**DOI:** 10.1371/journal.pone.0028658

**Published:** 2011-12-05

**Authors:** Caren C. Helbing, Mary J. Wagner, Katherine Pettem, Jill Johnston, Rachel A. Heimeier, Nik Veldhoen, Frank R. Jirik, Yun-Bo Shi, Leon W. Browder

**Affiliations:** 1 Department of Biochemistry & Microbiology, University of Victoria, Victoria, British Columbia, Canada; 2 Department of Biochemistry and Molecular Biology, University of Calgary, Calgary, Alberta, Canada; 3 Section on Molecular Morphogenesis, Laboratory of Gene Regulation and Development, Program on Cell Regulation and Metabolism, National Institute of Child Health and Human Development, National Institutes of Health, Bethesda, Maryland, United States of America; 4 The McCaig Institute for Bone and Joint Health, University of Calgary, Calgary, Alberta, Canada; Ecole Normale Supérieure de Lyon, France

## Abstract

**Background:**

INhibitor of Growth (ING) proteins belong to a large family of plant homeodomain finger-containing proteins important in epigenetic regulation and carcinogenesis. We have previously shown that *ING1* and *ING2* expression is regulated by thyroid hormone (TH) during metamorphosis of the *Xenopus laevis* tadpole. The present study investigates the possibility that ING proteins modulate TH action.

**Methodology/Principal Findings:**

Tadpoles expressing a *Xenopus ING2* transgene (Trans_ING2_) were significantly smaller than tadpoles not expressing the transgene (Trans_GFP_). When exposed to 10 nM 3,5,3′-triiodothyronine (T_3_), premetamorphic Trans_ING2_ tadpoles exhibited a greater reduction in tail, head, and brain areas, and a protrusion of the lower jaw than T_3_-treated Trans_GFP_ tadpoles. Quantitative real time polymerase chain reaction (QPCR) demonstrated elevated *TH receptor β* (*TRβ*) and *TH/bZIP* transcript levels in Trans_ING2_ tadpole tails compared to Trans_GFP_ tadpoles while *TRα* mRNAs were unaffected. In contrast, no difference in *TRα*, *TRβ* or *insulin-like growth factor* (*IGF2)* mRNA abundance was observed in the brain between Trans_ING2_ and Trans_GFP_ tadpoles. All of these transcripts, except for *TRα* mRNA in the brain, were inducible by the hormone in both tissues. Oocyte transcription assays indicated that ING proteins enhanced TR-dependent, T_3_-induced *TRβ* gene promoter activity. Examination of endogenous T_3_-responsive promoters (*TRβ* and *TH/bZIP*) in the tail by chromatin immunoprecipitation assays showed that ING proteins were recruited to TRE-containing regions in T_3_-dependent and independent ways, respectively. Moreover, ING and TR proteins coimmunoprecipitated from tail protein homogenates derived from metamorphic climax animals.

**Conclusions/Significance:**

We show for the first time that ING proteins modulate TH-dependent responses, thus revealing a novel role for ING proteins in hormone signaling. This has important implications for understanding hormone influenced disease states and suggests that the induction of ING proteins may facilitate TR function during metamorphosis in a tissue-specific manner.

## Introduction

ING proteins are implicated in the control of several key cellular processes including proliferation, apoptosis, DNA repair, senescence, and drug resistance; also their transcript levels are often reduced in cancer cells [1]–[3]. The latter property likely results from epigenetic regulation of the ING genes through events such as DNA hypermethylation, as mutations in these genes are rare [4]. Of the five known ING genes (*ING1-5*), *ING1* and *ING2* are most closely related to each other [5], [6]. Like all INGs, ING1 and 2 proteins belong to a large family of plant homeodomain (PHD) finger-containing proteins with a highly conserved Cys_4_-His-Cys_3_ motif, implying that these proteins regulate chromatin structure and hence gene expression [7]. Indeed, ING proteins have been shown to modulate transcription of genes involved in cell growth control and apoptosis [8] and they possess a consensus nuclear localization signal and a novel conserved region important in the interaction with histone acetyltransferases (HATs) and histone deacetyltransferases (HDACs) [9]. In addition to HAT/HDAC association, ING proteins interact with p53, transcription cofactors, and phosphoinositides [9], [10]. Genetic and crystal structure analyses have revealed that ING proteins bind to trimethylated lysine 4 of histone H3 (H3K4me3) in yeast and mammalian cells *via* their PHD domains [11]–[17]. H3K4me3 represents an epigenetic histone modification that is associated with gene promoter activation.

Considerable information exists regarding the steady-state levels of *ING* transcripts and proteins in a variety of tissues and cell lines. However, little is known about the regulation of *ING* expression and the contribution of ING proteins to developmental processes [18]. *ING* transcripts are differentially expressed in fetal *versus* adult human tissues [5], and their levels are particularly high in the brain of humans and frogs [5], [19]. Although not showing obvious signs of gross behavioral abnormalities, female *ING1* knockout mice showed a tendency to display an impaired ability to care for their young [20].

During tadpole metamorphosis into a juvenile frog, thyroid hormones (THs), such as 3,5,3′-triiodo-L-thyronine (T_3_), initiate the genetic programs for apoptosis, proliferation, and remodeling of tadpole tissues. Exogenous administration of TH to premetamorphic tadpoles induces precocious metamorphosis and facilitates investigation of TH-responsive pathways [21]. The mechanisms of TH action are highly conserved in vertebrates and are primarily through regulation of gene transcription *via* high affinity binding to specific nuclear TH receptors (TRs) that interact with TH response elements (TREs) located within the promoters of target genes [22]. We have previously shown that ING proteins are differentially expressed during postembryonic development of the *Xenopus laevis* tadpole [19], [23]. ING protein accumulated in serum-free tail organ cultures induced to undergo regression by T_3_ and this accumulation was prevented by inhibitors of tail apoptosis [19], [23].

The steady state levels of *Xenopus laevis ING1* and *ING2* transcripts change in a tissue-specific manner upon T_3_ treatment of premetamorphic tadpoles [19], [23]. Several transcript variants that we identified displayed increased levels in the tail (destined to undergo apoptosis), decreased levels in the hindlimb (destined to grow and proliferate), and relatively constant levels in brain (destined to undergo remodeling) [19], [23], [24]. Indeed, a molecular basis for the regulation of *ING1* and *ING2* transcripts by TH was recently elucidated when we characterized the *X. laevis* promoters of these genes; we discovered that they contained several putative TRE consensus sequences, and demonstrated differential promoter binding of TRs upon TH exposure [25].

*ING* genes not only represent targets for TH regulation, but they may also modulate the responses to hormone action. Toyama et al [26] found that p33^ING1b^ stimulated the transcriptional activity of the estrogen receptor α (ERα) in COS7 cells transfected with an estrogen-responsive reporter construct and an expression plasmid encoding human ERα. This stimulation appeared to be mediated through the AF2 site on ERα possibly *via* a direct interaction with ING [26]. The receptors for estrogen and thyroid hormone belong to the same protein superfamily and share extensive functional and sequence homology (for review see [22]). It is therefore plausible that p33^ING1b^ also regulates TH-mediated responses as well.

This study sets out to examine the role of ING during TH-dependent amphibian postembryonic development. We generated transgenic *Xenopus laevis* tadpoles overexpressing *ING2* and found that elevated levels of *ING2* promote TH-dependent metamorphosis and disrupt the TH-dependent gene expression responses. A combination of *Xenopus* oocyte injection, chromatin immunoprecipitation, and coimmunopreciptation assays demonstrate that

ING proteins associate with TRs and modulate TR-mediated gene transcription, thus revealing a novel role for ING proteins in TH-signaling during development.

## Materials and Methods

### Animal exposures

The care, use, and treatment of the amphibians in the present study were approved by the Animal Care Committees of the University of Victoria (protocol #2005-001) and the University of Calgary (protocol # 88043) under the auspices of the Canadian Council on Animal Care. *Xenopus laevis* tadpoles were purchased from Ward's Natural Science (St. Catherines, ON) and Xenopus I, Inc. (Dexter, MN) and maintained in polyethylene buckets at room temperature. Tadpoles were fed Seramicron daily (Rolf C. Hagen, Inc., Montreal, QC). Premetamorphic Nieuwkoop and Faber (NF; [27]) stages 52–54, *X. laevis* tadpoles were acclimatized to lab conditions at 21±1°C in polyethylene buckets and transferred to glass dishes with charcoal-filtered municipal water at 21±1°C with a density of ∼5 tadpoles per liter. After 24 h, during which time animals were not fed, either 10 nM T_3_ (Sigma-Aldrich) dissolved in 400 µM NaOH (EM Science) was added to the water for treated tadpoles or an equal volume of 400 µM NaOH was added to vehicle control tadpoles. Tadpoles were euthanized in 0.1% tricaine methanesulfonate (MS-222; Syndel Laboratories, Vancouver, BC) at 48 h post-treatment.

Transgenic tadpoles were either maintained in plastic food containers in which water was changed manually as needed or in continuously circulating Z-MOD tanks (Marine Biotech, Beverly, MA). After metamorphosis, frogs were maintained in Z-MOD tanks until they became large enough to be transferred to larger-sized X-MOD tanks.

### Preparation of the ING2 expression construct for transgenesis

Two approaches were taken to generate transgenic animals overexpressing *ING2* from the X. laevis gene (Trans_ING2_). In either case, the resultant plasmid contained a CMV driven ING2 gene and an independent cardiac actin promoter-driven green fluorescent protein (GFP) cassette. Transgenic animals were also generated with siblings using the plasmid construct containing the GFP cassette only as controls (Trans_GFP_).

The first *ING2* construct was created as follows. The BamHI to SmaI fragment containing the *X. laevis ING2* open reading frame from pTOPOIIXing2 was ligated into the BamHI to SnaBI sites of pCS2. The resultant plasmid, pCS2Xing2, was then digested with NotI and dephosphorylated with calf intestinal phosphatase. The NotI fragment containing the XCAR-GFP-SV40 polyA cassette from pXCARGFP, a generous gift from K. Kroll, was ligated into the NotI digested pCS2Xing2. The orientation of the XCARGFP cassette in the plasmid has not been determined. The resultant plasmid, pCS2Xing2XCARGFP, was linearized with KpnI prior to the generation of transgenic tadpoles.

An alternate strategy was also used where pET-Xing2 (described above) was digested with StyI and the ends of the gene (*ING2his*) fragment were filled with Klenow DNA polymerase. The gene fragment was then ligated into SmaI/dephosphorylated pBSK to make pBSK-Xing2his. pBSK-Xing2his was digested with BamHI and the ends filled in with the Klenow polymerase. A subsequent digest with EcoRI released the gene fragment which was ligated into EcoRI/SnaBI digested pCS2 to make pCS-Xing2his. The XCAR-GFP-SV40 polyA cassette was added to this plasmid at the NotI site as described above. Animals generated from either expression cassette were pooled together for analysis to increase sample size.

### Transgenesis

The transgenesis protocol employed was based upon the technique described by Sparrow [28], which is a simplification of the procedure originally described [29]. The simplified version eliminates the step that uses the egg extract and restriction enzyme. We have found no significant difference in the frequencies of either normal development, transgenesis or viability of embryos, tadpoles or frogs using this modification (data not shown). The capacitation reaction was done by mixing 2.5×10^5^ nuclei in 2.5 µl of sperm storage buffer and 100 ng linearized DNA in 2.5 µl of water followed by 10 min incubation at room temperature (∼20–23°C). At this point, 495 µl of sperm dilution buffer was added, and the suspension was mixed gently before loading into microinjection needles for nuclear transplantation. Embryos with transplanted nuclei were screened for transgene expression by detection of GFP fluorescence using an Olympus SZX9 fluorescence stereomicroscope. All experiments using transgenic animals utilized the F_0_ generation.

### Morphometric measurements

Dorsal and lateral digital images of chemically-treated animals were obtained prior to tissue preservation using a fixed-position Nikon Coolpix 5400 (5.1 megapixel) camera in macro mode with the flash turned off. Photographs of tadpoles included a ruler as an internal length standard. Images were collected at day 5 of exposure. Length measurements were determined in millimeters, whereas area measurements were collected in pixels using Photoshop Version 7.0 software (Adobe Systems Inc., San Jose, CA).

### Isolation of RNA and quantitative real-time polymerase chain reaction (QPCR)

Total RNA from tails and brains was isolated and converted to cDNA as described previously [30], [31]. A MX4000 real-time quantitative polymerase chain reaction system (Stratagene, La Jolla, CA) was used to examine the expression of several gene transcripts. The reactions were performed as previously described [30], [31] and the primers and annealing conditions are listed in Table 1. For each set of QPCRs, two controls were included to determine the specificity of target cDNA amplification: one without cDNA template and one without Taq DNA polymerase. The specificity of the appropriate products was verified by electrophoresis on 2% agarose gels stained with ethidium bromide and sequence confirmation of the resultant band. The thermocycle started at 95°C (9 min), followed by 40 cycles of: 95°C (15 sec), 30 sec annealing (see Table 1 for temperatures) and 72°C (45 sec) extension. Quadruplicate reactions were performed for each sample and cycle threshold (Ct) data obtained were compared to the geometric mean of normalizer gene transcripts indicated in Figure S1 using the comparative Ct method (ΔΔCt) (http://www.dorak.info/genetics/realtime.html). Normalizer gene transcripts were chosen based upon NormFinder (http://www.mdl.dk) and covariance (Cronbach's α) criteria. Amplification quality was monitored as described previously [19]. The resultant gene expression data are presented as fold change relative to the Trans_GFP_ control animals.

**Table 1:** QPCR primer sequences and annealing temperatures.

Gene	Primer Sequence(F: Forward; R: Reverse)	AnnealingTemperature(°C)
*Inhibitor of growth 2 (ING2)*	F: GAGTGCGTGGAGTCGTTGR: CTTGGCTTTGGAGCGTTT	58
*Ribosomal L8 (rpL8)*	F: AGAAAGGGTGCTGCTAAGR: GATGGGTTTGTCAATACG	55
*β-Actin*	F: TCACCACCACAGCCGAAAGR: GGGCCAGACTCATCATACTCCT	55
*β-Amyloid precursor*	F: CCCCTGACGCAGTTGACAR: CGGATTTGAGCCGCCTTC	55
*Thyroid hormone receptor g a (TRα)*	F: GGAGTGGGAGTTGATTCGCAR: CTTCCGTATCGTCAAGGTTA	55
*Thyroid hormone receptor β (TRβ)*	F: CACAAGAAGAATGGGAGTR: TCTGATGACATAAGCAGC	55
*Insulin-like growth factor-2 (IGF2)*	F: CACAGCAATACCACCACTR: TTCTTCAGCCTTCTCCAT	62
*TH-responsive basic leucine zipper transcription factor (TH/bZIP)*	F: CGTGTCATTGCCCTTCTTR: TGGTGGTACTCCGTCTTG	55

### In vitro synthesis of capped mRNA

The synthesis of mRNAs *in vitro* was performed using linearized DNA templates and a SP6 mMESSAGE mMACHINE kit (Ambion, Inc) as described by the manufacturer. The templates used for the reactions were pSP64(A)-TRβA [32], pSP64(A)-RXRα (heterodimeric binding partner of TR for maximal transcriptional activation [32]), *ING1* and *ING2* for synthesis of mRNAs encoding *Xenopus* TRβ, RXRα, ING1 and ING2. The capped mRNA was purified and resuspended in RNase-free water.

### Transcription assay in a *Xenopus* oocyte system

Microinjection experiments were performed using stage VI *Xenopus* oocytes, essentially as described by Wong and Shi [32]. Briefly, the cytoplasm of the oocytes was injected with the indicated mRNAs (1.15 ng/oocyte for TRβ and RXR, 1.15 to 2.3 ng/oocyte for ING1 and ING2), whereas the luciferase reporter plasmid TRE-Luc (0.33 ng/oocyte) and the control vector phRG-TK (0.03 ng/oocyte: Promega) were coinjected into the germinal vesicle (nucleus) following mRNA injection. The injected oocytes were incubated at 18°C in MBSH buffer [32] overnight in the presence or absence of 50 nM T_3_. For transcriptional analysis, a group of 20 oocytes was used for each sample to minimize the variation among oocytes and injections. After overnight incubation, the oocytes were assayed with a Dual-Luciferase® Reporter Assay System (Promega). Five oocytes were used for each luciferase assay; assays were performed in triplicate, and the experiments were repeated three times. The ratio of the relative expression of the firefly luciferase activity from the reporter plasmid to that from the control *Renilla* luciferase plasmid was determined for each assay group. Activity from the control *Renilla* luciferase was not affected by ING expression (data not shown). The averages from the repeated experiments were plotted together with the standard errors of the mean.

### Chromatin immunoprecipitation (ChIP) assays

Tadpoles were treated with T_3_ as described above, and DNA-protein complexes were isolated from tadpole tails. Five to six independent sets of pooled animals were used in ChIP assays where each pool contained cross-linked chromatin complexes from 7-12 tails using the method described in [25]. The precleared sonicates were adjusted to an appropriate volume such that 3 OD_260_ units were in 400 µl for each ChIP. This was then added to tubes containing 5 µl of rabbit polyclonal antibody (equivalent to 1 µg where known): either anti-TRα or anti-TRβ (gifts from D. Brown, Carnegie Institute, described in [33]); anti-ING (gift from K. Riabowol, University of Calgary); or a negative control antibody specific to herpes virus (6D9; Immuno-Precise Antibodies Ltd, Victoria, BC) as described in [25].

Analysis of ChIP DNA was done by PCR with primers specific for different promoter regions of interest along with control primers. PCR was done on a MX4000 thermocycler (Stratagene, La Jolla, CA). Primer design was accomplished using Primer Premier version 5 software (Premier Biosoft International) and primers synthesized by Qiagen. The *TRβ* and *TH/bZIP* promoter regions amplified were 203 bp and 284 bp, respectively using primers described by Havis *et al*. [34]. Primers specific for the *β-actin* gene were used as a negative control region for genomic DNA not associated with a promoter. The *β-actin* primers used were those published by Veldhoen *et al.*, 2002 (forward TCACCACCACAGCCGAAAG; reverse GGGCCAGACTCATCATACTCCT reverse primer) but were originally designed for amplification of *β-actin* mRNA of a 502 bp product in the open reading frame [35]. As the primers span exons 4 to 6 (GenBank accession number M24770), they produce a genomic DNA product of approximately 700 bp (determined empirically by agarose gel; data not shown). Although the input always produced a strong signal, ChIP with any antibody followed by amplification with the β-actin primers did not produce a signal above background (Figure 5C).

The PCR conditions for the primer sets for the *TRβ* promoter were: 9 min at 95°C and 40 cycles of 30 s at 95°C, 30 s at 60°C, and 30 s at 72°C. For the primer sets for *TH/bZIP*, the PCR conditions were: 9 min at 95°C and 37 cycles of 30 s at 95°C, 30 s at 60°C, and 30 s at 72°C. For the *β-actin* control, conditions were: 9 min at 95°C, and 34 cycles of 30 s at 95°C, 30 s at 62°C, and 30 s at 72°C. The PCR was done with 5 pmol primer (except *TH/bZIP* which was 2.5 pmol) in a 25 µl reaction with 10x reaction buffer (Invitrogen), 1.5 units of Platinum *Taq* DNA polymerase (Invitrogen), 10 nmol of dNTPs (GIBCO Life Technologies), and variable amounts of MgCl_2_ (1.5 mM for *TH/bZIP* and *β-actin* control, and 2 mM for TRβ).

The amplified products were separated on 2% agarose gels and visualized by ethidium bromide staining. To ensure comparability between gels, additional lanes containing the same quantity of a standard PCR product was run on each gel. The overall gel staining intensities were adjusted using these lanes. For a given primer and sample set, all samples were run together in the same PCR run, and, whenever possible, also run on the same agarose gel.

Densitometric analyses were performed using Northern Eclipse v5.0 (Empix Imaging Inc., Mississauga, ON). Densitometric values were local background subtracted and expressed as a percentage of the signal obtained from the input for each sample set.

### Statistical Analyses

Statistical analyses were conducted using PASW18 (SPSS, Chicago, IL, USA) and Systat 13 (SYSTAT Software, Inc., Chicago, IL, USA) software. Parametric data were analysed using ANOVA with the Tukey (equal variances) or Tamhane (equal variances not assumed) post hoc tests. Levine's test was used to determine whether data had equal variances or not. If the normality criterion using the Shapiro-Wilk test was not satisfied, then the non-parametric Kruskal-Wallis one-way analysis of variance was conducted followed by pairwise comparisons using the Mann-Whitney U test. Significance was set at p≤0.05.

### His-tagged proteins and antibodies

Western blot analyses were done using mouse monoclonal antibodies that were generated from purified bacterially-expressed *X. laevis* ING2-His and TRβ-His proteins (Immuno-Precise Antibodies Ltd., Victoria, BC). To generate His-tagged proteins for expression and purification from bacteria, appropriate vectors were generated. PCR primers were designed using Primer Premier version 5 software (Premier Biosoft International) such that amplicons for the open reading frame of either *ING1*, *ING2*, *TRα*, or *TRβ* would have cut sites for NcoI and XhoI restriction enzymes on either end. In order to accommodate the NcoI restriction enzyme sites in *ING1* and *ING2*, one bp was modified such that the first amino acid after the starting methionine would be a valine rather than a leucine. To accommodate the XhoI site and allow for coding of six histidine residues at the C terminal end of each protein, the stop codon was replaced with sequence coding for leucine and glutamate followed by six histidines. Primers were as follows: *ING1*: forward CGACGCCATGGTGAGCCCGGCAA, reverse GCTACCTCGAGCCTGTTATATGTCCT; *ING2*: forward CGACGCCATGGTAGGGCAACAGCAGCAC, reverse GGGTACCTCGAGCCTCGACCGTCTGTCTTT; *TRα* forward CGACGCCATGGACCAGAATCTAGCG, reverse GCTACCTCGAGAACTTCCTGGTCCTC; and *TRβ* forward CGACGCCATGGAAGGGTATATACCC, reverse GCTACCTCGAGGTCCTCAAACACTTCCAAG. Primers were used at 20 pmol in a typical 50 µl reaction containing 1.5 units of *Taq* DNA polymerase (Amersham Biosciences), 10 nmol dNTPs (GIBCO Life Technologies), and 1.5 mM MgCl_2_. PCR was done with adult testis cDNA as follows: 10 min 94°C denaturation, followed by 35 cycles of 94°C for 630 s, 55°C for 60 s and 72°C for 2 min, followed by 10 min at 72°C. PCR products were digested with NcoI and XhoI, gel-purified with the QIAEX II Gel Extraction Kit (Qiagen), and inserted into the pET21d^+^ vector (Novagen, Madison, WI). The sequence-verified plasmids were used to generate purified His-tagged protein using a column containing Talon metal affinity resin (Clontech, Palo Alto, CA) and sample integrity was verified using a 1/1000 dilution of an anti-His (H-15) rabbit polyclonal immunoglobulin (Ig) G as the primary antibody (Santa Cruz Biotechnologies Inc., Santa Cruz, CA) on immunoblots as described below.

The anti-ING 9H3 and anti-TR 9B2 antibodies were generated and their relative affinities and cross-reactivity to other His-tagged proteins were determined along with polyclonal TR mouse serum (taken from the mouse used for the creation of the TR monoclonal antibody). The polyclonal antibody was used in IP experiments whereas the anti-TR and anti-ING monoclonal antibodies were used in immunoblots. The anti-TR polyclonal antibody has a 5-fold greater preference for TRβ but also recognizes TRα and does not cross react with ING1 or ING2 (Figure S2). The anti-TR monoclonal (9B2) has a strong preference for TRβ, recognizing 200-fold higher amounts compared to TRα and does not cross react with ING1 or ING2. The anti-ING mouse monoclonal (9H3) recognizes both ING1 and ING2, with a 2- to 3-fold preference for ING2 and does not cross react with TRα or TRβ.

For chromatin immunoprecipitation assays, we used rabbit polyclonal antibodies anti-TRα or anti-TRβ (gifts from D. Brown, Carnegie Institute [33]); anti-ING (generated using a human GST-ING1 C-terminal end fusion protein which contains the highly conserved portion of ING [36]; a gift from K. Riabowol, University of Calgary); or a negative control antibody specific to herpes virus (6D9; Immuno-Precise Antibodies Ltd, Victoria, BC).

### Immunoblotting

Purified His-tagged proteins boiled in SDS sample buffer were subjected to electrophoresis through 7–17% or 12% SDS –PAGE gradient gels and immunoblotted as described previously [23] except 1/10 dilutions of tissue culture supernatants were used for the anti-frog TR (9B2) and anti-frog ING (9H3) antibodies. In addition anti-TR mouse polyclonal serum taken from the same mouse that was used to create the monoclonal anti-TR antibody was used at 1/1000 dilution.

After incubation with primary antibody, blots were washed and incubated with an IRDye 800CW conjugated anti-rabbit IgG secondary antibody with emission wavelength at 800 nm (Rockland Inc., Gilbertsville, PA) diluted 1∶2000 in 1% nonfat milk/phosphate buffered saline (PBS) with 0.15% Tween 20 (PBST) for 1 h at room temperature with shaking. The blots were then washed with PBST for 5×10 min followed by 2 final washes with PBS for 2×5 min. Alternatively, after incubation with the primary antibody, the blot was rinsed 6× for 10 min each with cold 1× TBST (20 mM Tris HCl, pH 7.5, 500 mM NaCl, 0.15% Tween 20), then incubated with the secondary antibody for 30 min in the dark at room temperature. Six 10 min rinses were then repeated in the dark at room temperature, followed by a final 3× wash with 1× TBS. The membrane was allowed to dry in the dark before scanning.

Blots were scanned at 42 µm resolution with an Odyssey infrared imaging system (LI-COR Biosciences, Lincoln, NE).

### Immunoprecipitations (IPs) with total protein from metamorphic climax tadpole tails

Tails from pools of 7 to 20 tadpoles during metamorphic climax, NF stage 60–62, were homogenized as described previously [19], [23]. Homogenates were centrifuged at 12,000xg for 10 min at 4°C and the collected supernatant was stored at −70°C. The homogenate concentration was determined using the BioRad Protein Assay according to the manufacturer's instructions (BioRad).

One mg of total protein was diluted to a 500 µl volume in IP buffer (50 mM HEPES, pH 8.0, 150 mM NaCl, 2.5 mM EGTA, 1 mM EDTA, 0.1% Tween-20) with 10 µM ZnSO_4_ added. Homogenates were precleared by rotation with 20 µl of protein G-Sepharose beads (Amersham) for 30 min at 4°C. The mixture was centrifuged at 3,000xg for 3 min at 4°C to pellet the beads, and the precleared protein solution was incubated overnight with 5 µl of anti-TR polyclonal mouse serum. Control IPs lacking protein homogenate or antibody were included. The following day, 20 µl of fresh beads were added to each IP. The antibody-bead homogenate mixture was incubated for 6 h at 4°C on an orbital shaker. Then the beads were washed and prepared for loading on SDS-PAGE gels as described above. Immunoblotting was done as described above using either anti-TR 9B2 or anti-ING 9H3 mouse monoclonal tissue culture supernatants for the primary antibody, each at 1/10 dilution. Each experiment was repeated three times with similar results. Binding specificity was determined by comparing immunoblots with unblocked anti-ING antibody to blots with anti-ING antibody that was preincubated with either 5 µg of purified ING1-His or ING2-His.

## Results

### ING2 transgenic tadpoles are smaller and show an altered response to TH-dependent metamorphosis

In order to ascertain the effect of ING2 overexpression on frog development, *Xenopus laevis* tadpoles were made to express an *ING2* transgene. The basic plasmid construct contains a *Xenopus* cardiac actin promoter (XCAR)-driven green fluorescent protein (GFP) that enables the rapid screening of embryos for positive transgenics by virtue of detectable GFP expression in mesoderm (data not shown). For the *ING2* transgenic animals, the GFP reporter cassette was ligated into a CMV promoter-driven *ING2* construct. Another construct was made in which the *ING2* open reading frame was modified at the C-terminus to include a six-His extension to the native protein. Since the responses were similar with either *ING2* construct, the data are presented combined as Trans_ING2_ animals. Finally, we used a control expression plasmid containing CMV promoter-driven *GFP* to ascertain if overexpression of a protein unrelated to *ING* has any effects. These animals are referred to as Trans_GFP_ animals.

**Figure 1:**
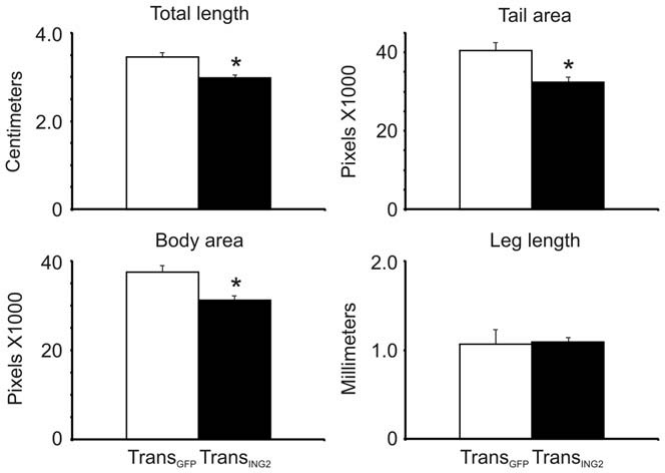
Comparison of morphological characteristics of *ING2*-overexpressing tadpoles (Trans_ING2_, filled bars; n = 19) and transgenic tadpoles expressing GFP only (Trans_GFP_, empty bars; n = 10). The asterisk denotes a significant difference between the two groups (ANOVA, p<0.001; body area: Mann Whitney U, p = 0.005). The error bars represent the standard error of the mean.

**Figure 2:**
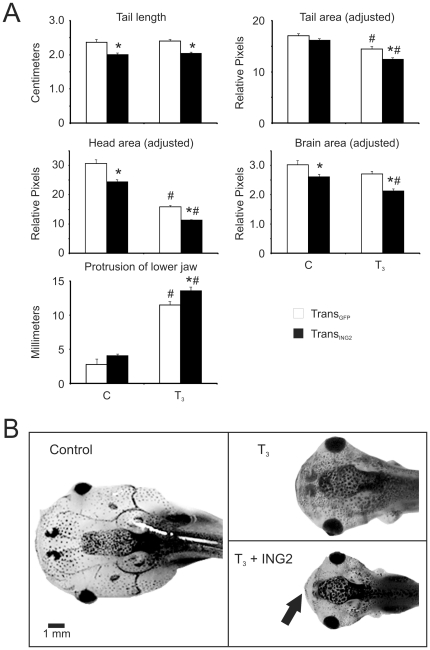
ING2 overexpression influences tadpole morphology upon T_3_ exposure. A) Comparison of morphological responses to T_3_ exposures of *ING2*-overexpressing tadpoles (Trans_ING2_, black bars; n = 20) and transgenic tadpoles expressing GFP only (Trans_GFP_, white bars; n = 10). Vehicle control animals (C) are compared with tadpoles treated with 10 nM T_3_ for 5 days (T_3_). The asterisk denotes a significant difference between the Trans_ING2_ and the Trans_GFP_ transgenic animals (ANOVA, p<0.04; brain area: Mann Whitney U, p = 0.033). The “#” indicates statistical significance relative to the vehicle control within a transgenic type. The error bars represent the standard error of the mean. “Adjusted” data were corrected for the differences in body sizes by dividing the area values by the tail lengths before analysis of T_3_-dependent effects. B) Dorsal view of representative *X. laevis* tadpoles that were exposed to vehicle control (“C”) or T_3_ for 5 days. The black arrow indicates a more prominent protrusion of the lower jaw in the Trans_ING2_ animals compared to Trans_GFP_ transgenic animals.

Elevated constitutive expression of the *ING2* transgene was not lethal to early developing embryos. However, these Trans_ING2_ tadpoles were significantly smaller (∼18%) according to total length, body and tail area than the Trans_GFP_ animals (Figure 1), although they did not develop at a different rate (e.g. leg length was the same, Figure 1). Tail length was 37% shorter in the Trans_ING2_ tadpoles (Figure 2A; compare Control white bars to black bars) and Trans_ING2_ tadpoles had significantly smaller head and brain areas compared to Trans_GFP_ animals (25 and 18%, respectively; data not shown).

Trans_GFP_ premetamorphic tadpoles immersed in water containing 10 nM T_3_ showed a significant reduction in tail area (15%) and head area (48%) and 4-fold increase in the protrusion of the lower jaw (Figure 2A; white bars). Brain area was moderately affected (10% reduction) and tail length was unaffected by hormone treatment (Figure 2A; white bars).

Morphometric data was corrected for difference in sizes between Trans_ING2_ and Trans_GFP_ animals by using the tail lengths before comparing the animals' responses to T_3_ treatment. The T_3_-induced reductions in tail, head, and brain area and the protrusion of the lower jaw seen in the Trans_GFP_ animals were all significantly enhanced in the Trans_ING2_ animals (Figure 2A; compare T_3_ white bars to black bars, and Figure 2B). Not all morphological changes induced by T_3_ were different between the Trans_GFP_ and Trans_ING2_ animals. The induced reduction in body area (32%) and body length (24–27%) and an increase in hindlimb length (∼2 fold) were indistinguishable between the Trans_GFP_ and Trans_ING2_ animals (data not shown). These observations suggest that certain tissue types are more sensitive to *ING2* overexpression.

**Figure 3:**
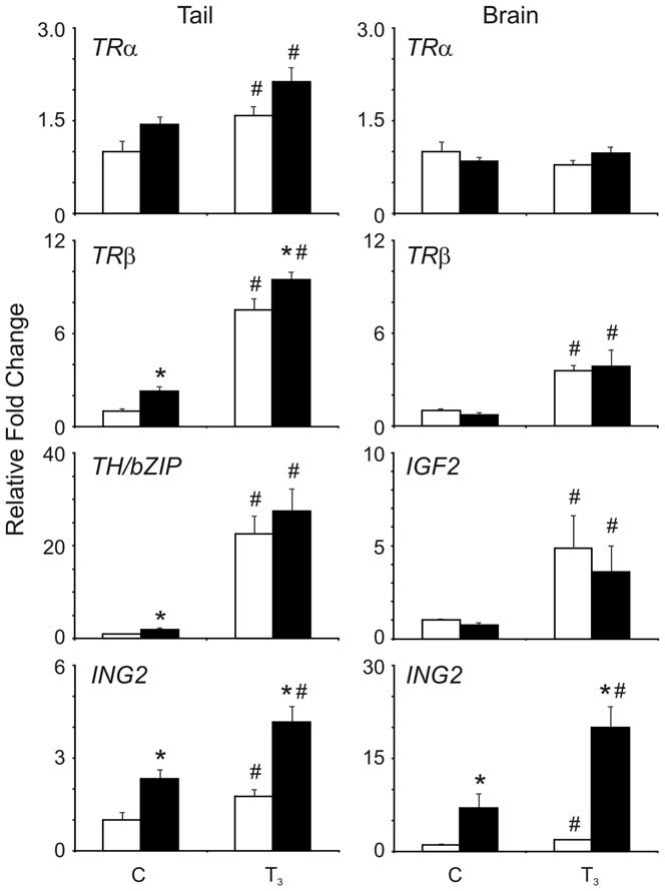
QPCR data of mRNA isolated from the tails and brains of Trans_GFP_ (white bars; n = 5) or Trans_ING2_ (black bars; n = 9–10) transgenic tadpoles treated with solvent only (C) or 10 nM T_3_ (T_3_) for 48 h. The gene transcripts are indicated above each graph. The bars denote the relative transcript levels derived as described in the Materials and Methods. The asterisk denotes a significant difference between the Trans_ING2_ and the Trans_GFP_ transgenic animals (p<0.05). The “#” indicates statistical significance relative to the vehicle control within a transgenic type.

Given that regulation of mRNA expression is a major mechanism of TH action and that ING proteins are known to associate with chromatin and modulate gene promoter activity, we examined whether *ING2* overexpression affects the TH-mediated developmental program in transgenic animals. QPCR data from tail and brain samples collected from Trans_ING2_ and Trans_GFP_ tadpole tails showed that Trans_ING2_ tadpoles expressed 2- and 7-fold higher *ING2* transcripts compared to Trans_GFP_ tadpoles (p = 0.01, tail; p = 0.03, brain; Figure 3; compare Control white bars to black bars). Exposure of Trans_GFP_ animals to T_3_ resulted in a significant induction of *ING2* transcript levels in both tissues as expected from previous work [19] and the T_3_-dependent induction was more pronounced in the Trans_ING2_ animals (p = 0.05 and 0.008, tail; p = 0.01 and 0.01, brain).

We examined the levels of *TRα*and *TRβ* mRNAs in both tissues as well as *TH/bZIP* and *insulin-like growth factor 2 (IGF2)* known to be up-regulated by TH, in tail and brain, respectively [37]. As expected, these transcripts increased in abundance upon T_3_ treatment while *TRα* mRNA levels were unchanged in the brain (Figure 3 white bars; *TRα*: p = 0.04, tail; *TRβ*: p = 0.0001, tail; p = 0.003, brain; *TH/bZIP*: p = 0.001, tail; *IGF2*: p = 0.05, brain). Overexpression of the *ING2* transgene resulted in a significant elevation of *TRβ* and *TH/bZIP* mRNAs in the tail of Trans_ING2_ animals compared to Trans_GFP_ animals in the control condition (Figure 3; *TRβ*: p = 0.03; *TH/bZIP*: p = 0.02) whereas *TRα* mRNAs remained marginally unaffected (Figure 3; *TRα*: p = 0.06). Upon T_3_ treatment, a significant difference between the Trans_ING2_ and Trans_GFP_ animals for *TRβ* transcripts, but not for *TRα* and *TH/bZIP* mRNAs, was observed (Figure 3; *TRα*: p = 0.09; *TRβ*: p = 0.05; *TH/bZIP*: p = 0.47). *TRα*, *TRβ*, and *IGF2* transcripts in the brain were not affected by *ING2* overexpression in either treatment condition (Figure 3).

### ING proteins enhance TR activity

**Figure 4:**
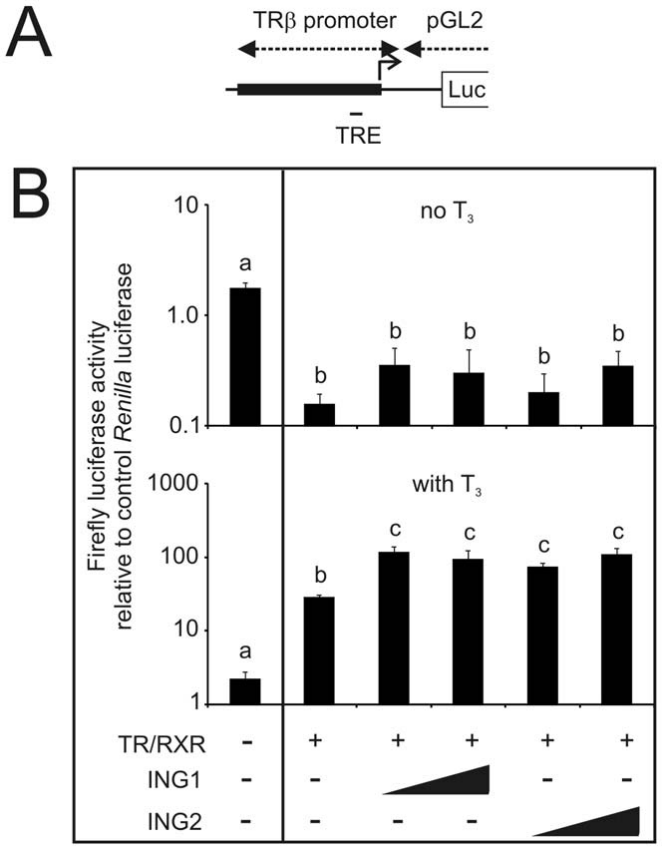
ING proteins enhance the ability of TRâ to activate transcription in the presence of T_3_ in a *Xenopus* oocyte transcription system. Microinjection experiments were performed using stage VI *Xenopus* oocytes. The oocyte cytoplasm was injected with the indicated mRNAs (1.15 ng/oocyte for TR and RXR, 1.15 or 2.3 ng/oocyte for ING1 and ING2). Following mRNA injection, all oocytes were injected in the germinal vesicle with the luciferase reporter plasmid TRE-Luc (0.33 ng/oocyte) which has the T_3_-dependent TRâpromoter containing a TRE driving the expression of firefly luciferase (“pGL2” shown in “A”), and the control vector phRG-TK driving the control *Renilla* luciferase (0.03 ng/oocyte: Promega). B) The injected oocytes were incubated at 18°C overnight in the presence or absence of 50 nM T_3_. The oocytes were assayed with a Dual-Luciferase® Reporter Assay System (Promega). Plotted are the averages and the standard errors of the mean firefly luciferase activity relative to the *Renilla* luciferase activity. These results are from two experiments each done in triplicate. The error bars represent the standard error of the mean, and statistical significance of data relative to each other (p<0.05, Mann Whitney U) is indicated by different letters. Bars with the same letters are not statistically different from each other. Note that the scales of the two graphs are different.

The above results suggest that ING2 status is associated with modulation of TH activity in the frog tadpole. ING1 and ING2 proteins are closely related and both have the ability to affect chromatin structure [5], [6]. To begin to determine how ING proteins modulate the activity of the TR complex in TH-dependent gene transcription, we used an oocyte transcription system [38]. The *Xenopus* oocyte has an extensive store of basal transcription factors and histones in preparation for chromosomal assembly during early embryonic development. Functional analyses of chromatinized promoters driving a reporter gene are easily performed by microinjection of either purified regulatory proteins or their mRNA into the oocyte cytoplasm in addition to a plasmid construct containing a promoter and reporter gene injected into the nucleus where it can be assembled into chromatin [39]. The end result is an appropriately chromatinized promoter region. *Xenopus* oocytes have very low levels of endogenous TRs and coinjection of TRs with RXRs effectively represses basal transcription of a TRE/reporter construct (Figure 4). This repression is released upon further treatment of the oocyte with T_3_ [32] (Figure 4). We examined the effect of p33^ING1^ and p33^ING2^ on the basal and T_3_-inducible activity of the *TRβ* promoter as measured by firefly luciferase activity. A schematic of the promoter region construct is shown in Figure 4A. While neither ING protein affected basal transcription of the promoter (top of Figure 4B), both enhanced TR-associated promoter activity in the presence of 50 nM T_3_ (bottom of Figure 4B).

### ING proteins associate with promoter regions of TH-responsive genes

**Figure 5:**
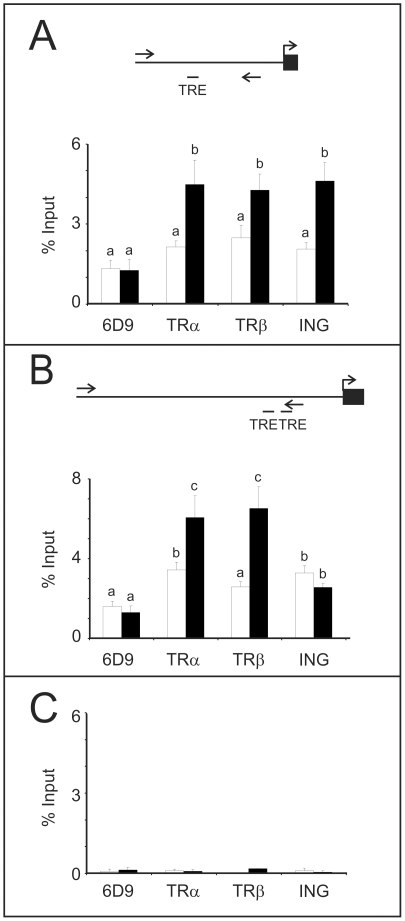
Chromatin immunoprecipitation assays indicate that ING protein associates with the *TRβ* and *TH/bZIP* promoters that are known to be T_3_-regulated in tadpole tails. The promoter regions amplified upon ChIP are indicated by the arrows in the cartoons above the graphs for A) *TRβ* and B) *TH/bZIP* promoters and C) a *β-actin* gene control. The graphs show the percent input values of the amplicons obtained after immunoprecipitation with the indicated antibodies directed against TRα, TRβ, ING, and a control antibody, 6D9. DNA-protein complexes were obtained from pools of 7–12 tails from tadpoles NF stage 52–54 that were either time-matched solvent controls (white bars) or treated with 10 nM T_3_ for 48 h (black bars). Independent sets of animals were tested and averaged (n = 3–6, *TRβ* promoter; n = 3–5, *TH/bZIP* promoter). The error bars represent the standard error of the mean, and statistical significance (p<0.05, Mann Whitney U) is indicated by different letters.

We then examined whether ING proteins are found associated with regions of TH-responsive genes containing TREs *in vivo*. Chromatin immunoprecipitation (ChIP) analyses were performed using extracts from tails of premetamorphic tadpoles induced with 10 nM T_3_ to undergo precocious metamorphosis or treated with vehicle alone. Antibodies against ING proteins showed association with a 203 bp region of the *TRβ* promoter and a 284 bp region of the *TH/bZIP* promoter; both of which contain TREs [40] (top of Figures 5A and B). ChIP assay results using anti-TR antibodies are consistent with previously published observations [41]. ChIP analyses of the constitutively expressed *β-actin* gene showed no association of ING proteins demonstrating that the observed associations were specific to the *TRβ* and *TH/bZIP* promoters (Figure 5C). A T_3_-dependent recruitment of ING proteins was detected on the *TRβ* promoter (Figure 5A), whereas the *TH/bZip* promoter showed constant association with ING proteins (Figure 5B). The anti-ING antibody used to generate these data recognizes both p33^ING1^ and p33^ING2^ isoforms: therefore, we cannot discount the possibility of individual isoforms differentially associating with these promoter regions dependent upon TH levels.

### ING proteins coimmunoprecipitate with TRs

**Figure 6:**
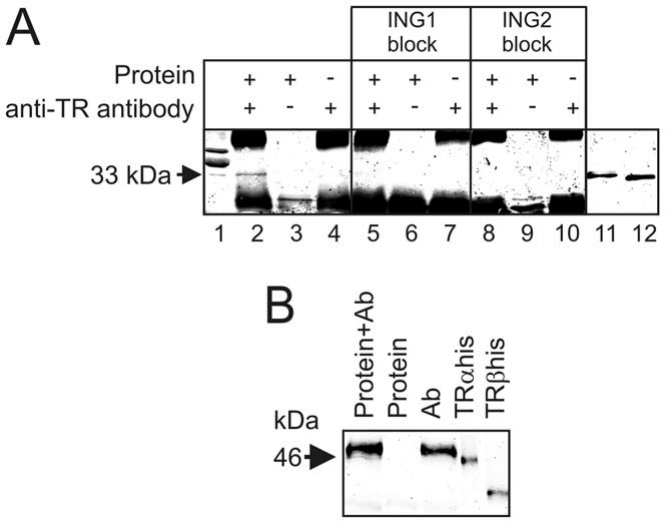
Evidence that ING proteins are present in TR-containing complexes. A) Endogenous p33^ING^ protein coimmunoprecipitates with TRs in tadpole tail homogenates. Immunoprecipitations with a mouse polyclonal anti-TR antibody were carried out on *X. laevis* total tail homogenates from metamorphic climax NF stage 60–62 tadpoles (where ING, TH, and TR levels are all high), followed by immunoblots with a mouse monoclonal anti-ING antibody. A weak, but highly reproducible, specific 33 kDa band (indicated by an arrow) was detected (lane 2) when Sepharose beads were coincubated with anti-TR antibody and tail homogenate (Protein) and not present when either was incubated alone with the beads (lanes 3–4). Lane 1, protein homogenate (30 µg). This band is also not visible in Western blots where the anti-ING antibody was preincubated with either purified, bacterially-expressed p33^ING1^ (ING1 block; lanes 5–7) or p33^ING2^ (ING2 block; lanes 8–10). Lanes 11 and 12 are bacterially-expressed His-tagged *Xenopus* p33^ING1^ and p33^ING2^ proteins, respectively. Neither of the two slower migrating ING isoforms in lane 1 (see [49]) was immunoprecipitated. The strong upper and lower bands observed in lanes 2-10 are light and heavy chain immunoglobulins. B) Immunoblots of the same samples as above using another anti-TR antibody to demonstrate that TRs are immunoprecipitated. The 9B2 anti-TR antibody identified a band at ∼46 kDa that comigrates with bacterially-expressed frog TRα-His protein. The results shown represent one of three experiments with similar results.

To determine if TR and ING proteins are present in the same complex, we performed immunoprecipitations of TR-containing complexes followed by Western blotting of the immunoprecipitate with the anti-ING antibody using tail tissue lysate from metamorphic climax tadpoles. TR and ING proteins are expressed at high levels in the *X. laevis* tadpole tail at metamorphic climax [23], [33] and TH levels are also highest at this time [42]. We reproducibly identified a single 33 kDa protein band that comigrated with a protein recognized by the anti-ING antibody in total tail homogenate (Figure 6A). The band was specific for ING protein as it was not observed when beads were incubated with protein alone or with antibody alone (Figure 6A). Nor was it observed after blocking the anti-ING antibody with bacterially-expressed p33^ING1b^ or p33^ING2^ protein (Figure 6A). Successful immunoprecipitation of TRs was confirmed by Western blot of the same immunoprecipitates with a different anti-TR antibody (Figure 6B).

## Discussion

Our previous work showed that the levels of *ING* mRNA transcripts and protein increase in response to T_3_ treatment in the tadpole tail undergoing TH-dependent apoptosis [19], [23]. Herein we establish for the first time that ING proteins actively modulate the TH-mediated response *in vivo* and *in vitro*. Several lines of evidence indicate that this is through interaction with the TR-associated transcriptional complex, as ING proteins coimmunoprecipitate with TRs from tail homogenates, alter the TRE-mediated activity of a reporter construct in oocytes, and are found to associate with TRE-containing promoter regions in the tadpole tail.

TRs belong to a larger superfamily of nuclear receptors with common structures. The fact that *in vitro* translated p33^ING1b^ can weakly associate with the related estrogen receptor, ERα, and modulate transcription of an ER-responsive reporter gene in African green monkey kidney cells transfected with p33^ING1b^ [26] lends further support to our observations and suggests that ING proteins may utilize a mechanism of action that involves factors common to the regulation of multiple hormone signaling pathways. Misregulation of estrogenic and TH-dependent pathways is of particular interest in disease states such as cancer and obesity [43]–[48]. Therefore the present work showing the first biologically-relevant interaction of ING and TR proteins reveals an important consideration in these disease states; the relationship of ING levels to receptor activity. Moreover, since ING expression is controlled during development [49]–[51], ING/TR interplay is a plausible “fine-tuning” mechanism for tissue-specific responses in which induction and/or recruitment of ING proteins may enhance TR function.

The influence of ING on gene expression appears to be tissue-specific as was demonstrated for TR gene transcripts in the tail and brain (see Figure 3). ING2 overexpression increased the levels of both *TRβ* and *TH/bZIP* mRNAs in the tail but not the brain (Figure 3) and the maintenance of ING-enhanced levels of *TRβ* mRNAs upon T_3_ induction are consistent with the acceleration of T_3_-induced apoptosis observed in the tail of transgenic animals. A similar augmentation of *TRβ* mRNA levels was not observed within the brain, which may reflect the more moderate effect of ING overexpression on T_3_ induction in this organ. It is possible that there are other gene targets that may be influenced by ING overexpression in the tadpole brain that would contribute to the morphological outcome or that the transcript effects were not captured at the 48 h time point evaluated in this experiment. Microarray or RNA-seq approaches would help to address this issue. From our morphological observations, targetting specific brain regions for analysis rather than the whole brain, as in the present study, may better identify transcripts that are sensitive to ING2 overexpression in this organ. Moreover, transcriptomic analysis of the craniofacial region, which was affected by ING2 overexpression, is also warranted to further identify tissue-specific mechanisms.

TR-mediated transcription is an integral part of the tadpole metamorphic program. The intriguing observation that ING can associate with TR-containing complexes in tails undergoing apoptosis during metamorphic climax not only suggests that ING is involved in postembryonic development, but provides a mechanism for this involvement. ING family members possess no known enzymatic activity. It is therefore likely that ING proteins act by facilitating specific protein-protein and possibly protein-DNA interactions resulting in changes in the cofactor complement of TRE-associated complexes at specific TH-responsive promoters. ING coimmunoprecipitates with many proteins involved in DNA regulation, including p53, NF-κB, and PCNA, as well as HAT and HDAC chromatin remodeling factors (reviewed in [52]). TR itself binds gene promoter regions and can associate with proteins that interact with ING such as HAT, p300 and p53 (reviewed in [22]). It will be interesting to investigate further which proteins are associated with functional ING/TR complexes and under what conditions, particularly in response to changes in TH levels.

Whether ING interacts with TRs directly or indirectly is currently enigmatic. Specific isoforms of ING and TR may be involved in modulation of the regulatory response, providing a mechanism for promoter-specific effects. Moreover, the different effects of *ING2* overexpression on *TRβ* transcript levels in the tails compared to the brain upon T_3_ treatment suggest that tissue context contributes to the cellular effects of ING2 protein (see Figure 3). Even within the same tissue, genes containing TREs do not recruit ING proteins in the same way (Figure 5), nor do their transcripts respond to ING2 overexpression in the same way (compare *TRβ* and *TH/bZIP*; Figure 3). The presence of ING proteins on the *TH/bZip* promoter before T_3_-treatment may contribute to its order of magnitude higher responsiveness to T_3_ compared to a T_3_-dependent recruitment observed on the *TRβ* promoter (Figure 5). This overall enhancement role of ING on T_3_-induced transcription is consistent with the oocyte injection experiment results (Figure 4). Further analysis of the impact of ING recruitment to the promoter as well as TRE context, genetic and/or epigenetic, is needed.

It has been suggested that p33^ING1^ can compete for binding to an AT-rich sequence that is also an HNF-1 binding site as exemplified in the promoter for the α-fetoprotein gene [53]. In contrast, other work from our laboratory has failed to show any increased association of ING proteins with regions containing the consensus HNF-1 binding site *versus* regions lacking this site [25]. It therefore seems more likely that ING associates indirectly with DNA through other chromatin-associated proteins such as post-translationally modified histones such as H3K4 [11]–[13], [54]. Recent evidence also showed that the PHD finger of ING2 is capable of arresting haematopoietic differentiation and inducing leukemia [55] suggesting that this motif regulates developmentally important genes through interaction with H3K4me3.

Peptide binding assays indicated that ING proteins, via their PHD finger, bind to H3K4me3 and there is limited evidence that dimethylated H3K4 (H3K4me2) could also be a target, albeit with lower affinity [11]–[17]. Recent work using extensive ChIP analyses of modified histones on *TRβ* and *TH/bZIP* promoter regions in *Xenopus tropicalis* tail fins and brains [56] indicated that the TRE-containing region of *TRβ* had higher relative levels of H3K4me2/3 compared to the TRE-containing region of the *TH/bZIP* promoter [56]. T_3_ treatment did not affect H3K4me3 levels for either gene, but did result in differential recruitment of H3K4me2. Relatively high levels of H3K4me2 at the *TRβ* promoter decreased whereas the relatively low levels of this modified histone at the *TH/bZIP* promoter increased [56]. The observed ING protein recruitment patterns in the present study are not easily superimposed upon the H3K4me2/3 results reported in [56]. Assuming that *X. tropicalis* and *X. laevis* results are comparable, it is possible that the ratio of these methylated histones could contribute to differential ING recruitment that we observed in the present study. T_3_ treatment could alter this ratio, but also tissue-specific differences in absolute methylated histone levels may be contributory factors. This is merely speculative however, and much more investigation is needed within the same system to establish a relationship between H3K4 methylation status and ING recruitment at TH-responsive promoters. Recent work on HeLa cells represents an intriguing approach for identifying enrichment of nuclear factors on modified histones [54]. SILAC-based nucleosome affinity purifications and proteomics analyses on reconstituted recombinant nucleosomes and nuclear HeLa cell extracts did not identify ING1 or 2 proteins associated with H3K4me3, but did identify ING4 [54]. Unfortunately, this work was not done to examine TH responsiveness. It is important to note that the affinity of ING proteins for H3K4me2/3 in the context of native chromatin and a TH-responsive cell type has yet to be established.

In summary, multiple lines of evidence establish that TH-mediated gene expression during *Xenopus* postembryonic development can be modulated by ING proteins. The relative contributions of this important chromatin modulator to TR complex composition and activity, particularly with respect to tissue-specific responses, remain to be determined as do the effects of ING proteins on specific TH-responsive gene targets.

## Supporting Information

Figure S1 Expression levels of normalizer gene transcripts used in the present study. *Ribosomal protein L8* (*rpL8*), *β-actin* and *β amyloid precursor* mRNA QPCR data from the tails and brains of Trans_GFP_ (white bars; n = 5) or Trans_ING2_ (black bars; n = 9–10) transgenic tadpoles treated with solvent only (C) or 10 nM T_3_ (T_3_) for 48 h. The asterisk denotes a significant difference between the Trans_ING2_ and the Trans_GFP_ transgenic animals (p<0.05). The “#” indicates statistical significance relative to the vehicle control within a transgenic type. Cronbach's α for each tissue normalizer set indicating the degree of covariance is indicated.
(PDF)

Figure S2 Demonstration of antibody specificity of antibodies generated against purified His-tagged *X. laevis* p33^ING2^ and TRβ. To determine the specificity of these antibodies, samples of bacterially- expressed, Talon column-purified His-tagged proteins were separated by SDS-PAGE and immunoblotted with either a mouse monoclonal anti-TR antibody (9B2) or a mouse monoclonal anti-ING antibody (9H3). The sizes of the proteins are as follows: TRα-His ∼45 kDa, TRβ-His ∼37 kDa, ING1-His doublet ∼33 kDa and ∼35 kDa, and ING2-His ∼32 kDa. One μg protein was loaded in each lane except only 5 ng TRβ-His were used in the corresponding lane. 9B2 recognizes both TRα and TRβ with preference to TRβ, while 9H3 antibody recognizes both ING1 and ING2 proteins with preference to p33^ING2^.
(PDF)
