# Supplementary figures and images for: Modulation of Thyroid Hormone-Dependent Gene Expression in Xenopus laevis by INhibitor of Growth (ING) Proteins
Source: PLoS One. 2011 Dec 5;6(12):e28658. doi: 10.1371/journal.pone.0028658 (PMC3230625; doi:10.1371/journal.pone.0028658)

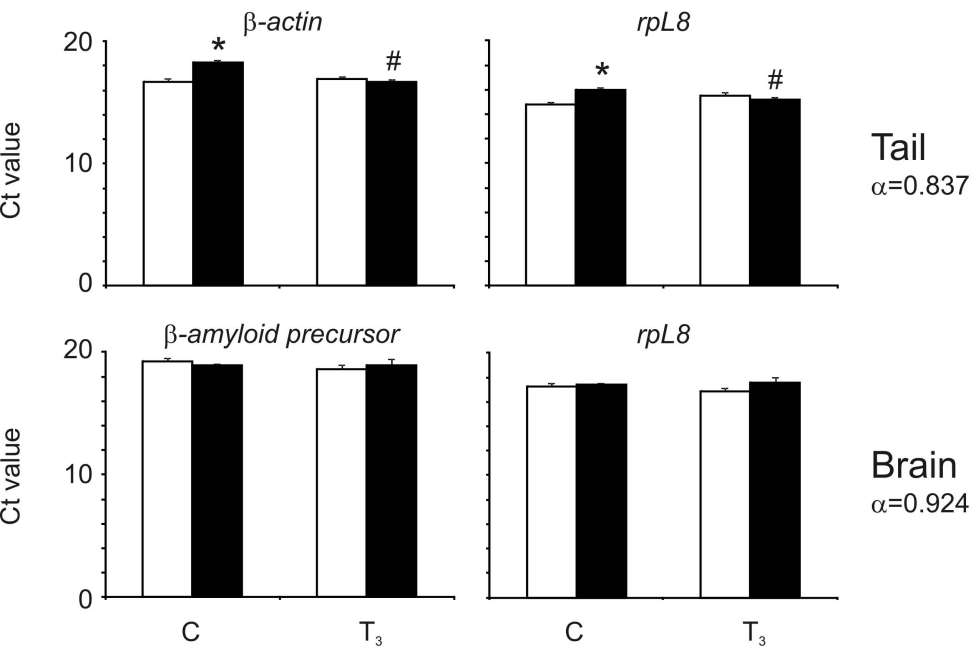

Supplement: Figure S1 — Expression levels of normalizer gene transcripts used in the present study. Ribosomal protein L8 (rpL8), β-actin and β amyloid precursor mRNA QPCR data from the tails and brains of TransGFP (white bars; n = 5) or TransING2 (black bars; n = 9–10) transgenic tadpoles treated with solvent only (C) or 10 nM T3 (T3) for 48 h. The asterisk denotes a significant difference between the TransING2 and the TransGFP transgenic animals (p<0.05). The “#” indicates statistical significance relative to the vehicle control within a transgenic type. Cronbach's α for each tissue normalizer set indicating the degree of covariance is indicated. (PDF) [file pone.0028658.s001.pdf]

Figure S2

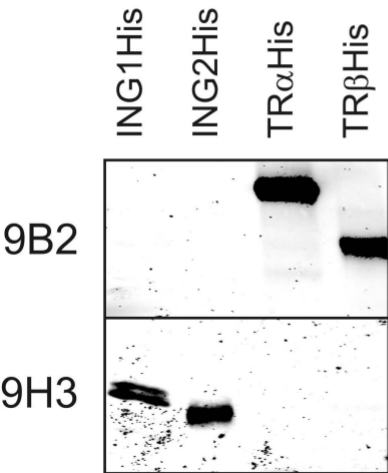

Supplement: Figure S2 — Demonstration of antibody specificity of antibodies generated against purified His-tagged X. laevis p33ING2 and TRβ. To determine the specificity of these antibodies, samples of bacterially- expressed, Talon column-purified His-tagged proteins were separated by SDS-PAGE and immunoblotted with either a mouse monoclonal anti-TR antibody (9B2) or a mouse monoclonal anti-ING antibody (9H3). The sizes of the proteins are as follows: TRα-His ∼45 kDa, TRβ-His ∼37 kDa, ING1-His doublet ∼33 kDa and ∼35 kDa, and ING2-His ∼32 kDa. One μg protein was loaded in each lane except only 5 ng TRβ-His were used in the corresponding lane. 9B2 recognizes both TRα and TRβ with preference to TRβ, while 9H3 antibody recognizes both ING1 and ING2 proteins with preference to p33ING2. (PDF) [file pone.0028658.s002.pdf]
